# The relationship between recreational cannabis use, psychotic-like experiences, and the salience network in adolescent and young adult twins

**DOI:** 10.1017/S0033291725101773

**Published:** 2025-10-07

**Authors:** Hande Atmaca-Turan, Didenur Şahin-Çevik, Serenay Çakar, Fulya Gökalp-Yavuz, Martijn van den Heuvel, Fruhling Rijsdijk, Francesca Filbey, Timothea Toulopoulou

**Affiliations:** 1 Institute for Diabetes Research and Metabolic Diseases of the Helmholtz Center Munich, The University of Tübingen, Tübingen, Germany; 2 Neuroscience Department, Bilkent University, Ankara, Turkey; 3 National Magnetic Resonance Research Center (UMRAM), Aysel Sabuncu Brain Research Centre (ASBAM), Bilkent University, Ankara, Turkey; 4 Department of Psychiatry and Psychotherapy, Central Institute of Mental Health, Medical Faculty Mannheim, Heidelberg University, Mannheim, Germany; 5 Department of Statistics, Middle East Technical University, Ankara, Turkey; 6 The Data Mine, Purdue University, West Lafayette, IN, USA; 7 Department of Complex Traits Genetics, Center for Neurogenomics and Cognitive Research, Vrije Universiteit Amsterdam, Amsterdam Neuroscience, Amsterdam, The Netherlands; 8 Department of Child Psychiatry, Amsterdam UMC, Amsterdam Neuroscience, Amsterdam, The Netherlands; 9 Psychology Department, Faculty of Social Sciences, Anton de Kom University of Suriname, Paramaribo, Suriname; 10 Department of Psychology, School of Behavioral and Brain Sciences, University of Texas at Dallas, Richardson, TX, USA; 11 Department of Psychology, Bilkent University, Ankara, Turkey; 12 First Department of Psychiatry, National and Kapodistrian University of Athens, Athens, Greece; 13 Department of Psychiatry, Icahn School of Medicine at Mount Sinai, New York, NY, USA

**Keywords:** adolescence, graph theory, psychotic-like experiences, recreational cannabis use, salience network, twin modeling

## Abstract

**Background:**

The use of cannabis in adolescence and early adulthood, critical phases for brain development, is linked to psychotic-like experiences (PLEs). The underlying mechanisms, however, remain unclear. This research examined the relationship between recreational cannabis use and PLEs, emphasizing the connectivity of the salience network (SN), which plays a role in salience processing and psychosis. To determine whether this relationship reflects shared genetic or environmental contributions, twin modeling was used.

**Methods:**

We included 232 healthy adolescent Turkish twins who underwent diffusion MRI and psychometric assessment. SN connectivity was quantified using graph theory metrics. Linear mixed models were used to examine the associations among cannabis use, SN factors, and PLEs. Mediation analyses assessed whether SN parameters explained the cannabis–PLEs association. Twin models disentangle genetic and environmental contributions to these traits and their covariation.

**Results:**

Cannabis use was significantly associated with higher overall PLE frequency. A specific SN factor predicted both total and positive PLEs. However, SN connectivity did not mediate the cannabis–PLEs relationship. Twin modeling showed that cannabis use and PLEs were mainly influenced by unique environmental factors. No significant phenotypic covariations were found among cannabis use, PLEs, and SN parameters.

**Conclusions:**

Recreational cannabis use during adolescence and young adulthood is associated with heightened PLEs, although this association is not mediated by SN connectivity. The environment plays an important role during adolescence in shaping these traits independently. The findings underscore the need for longitudinal and genetically informed studies to clarify the mental health effects of adolescent cannabis use.

## Background

With changing sociopolitical perspectives and legalization of cannabis in 22 countries and more than 30 U.S. states (Ferland & Hurd, 2020), recreational cannabis use has expanded. According to the 2020 European Drug Report, around 90 million people aged 15–64 have used cannabis at least once, with chronic users often beginning during adolescence (NIDA, 2020).

Adolescence, a key phase for brain development, is when cannabis exposure emerges as a psychosis risk factor (Blest-Hopley, Colizzi, Giampietro, & Bhattacharyya, 2020; Griffith-Lendering et al., 2013; Shrivastava, Johnston, Terpstra, & Bureau, 2014). Recently, it has been linked to psychotic-like experiences (PLEs), which resemble the symptoms of psychosis but are less severe in terms of frequency and disruption (Marconi, Di Forti, Lewis, Murray, & Vassos, 2016; Schubart et al., 2011; Sideli, Quigley, La Cascia, & Murray, 2020). Although often transient in youth, PLEs may precede a psychotic disorder, particularly when frequent. As recreational cannabis use rises, monitoring its impact on these experiences is increasingly important.

The causal relationship between cannabis and psychosis risk is increasingly recognized. A recent study shows that cannabis consumption increases the probability of psychosis, regardless of the polygenic risk for schizophrenia (Austin-Zimmerman et al., 2024). However, the neurobiological mechanisms underlying this association are still unclear. The endocannabinoid system is crucial during adolescence, and disruptions caused by THC lead to enduring neurobiological changes that affect behavior and function of the brain (Shrivastava et al., 2014). Neuroimaging studies reveal network-level abnormalities throughout the psychosis spectrum (Schmidt et al., 2015). Other neuroimaging studies, including those using diffusion tensor imaging (DTI), have associated adolescent cannabis use with alterations in white matter (WM) microstructure and brain network architecture (Bava et al., 2009; Courtney et al., 2022; Kim et al., 2011; Orr, Paschall, & Banich, 2016; Zalesky et al., 2012). Initial findings indicate that abnormal salience processing may connect cannabis consumption to PLEs (Dawes et al., 2022; Wijayendran, O’Neill, & Bhattacharyya, 2018). Cannabis users exhibiting elevated PLEs demonstrate reduced latent inhibition, suggesting challenges in filtering extraneous stimuli and a heightened likelihood of experiencing psychotic-like phenomena. The aberrant salience theory claims that abnormal dopamine transmission in the salience network (SN) leads to the inaccurate salience attribution to neutral stimuli, therefore contributing to psychotic symptoms (Kapur, 2003). Δ9-tetrahydrocannabinol (THC) has been linked to decreased connectivity in the SN, specifically between the insula and anterior cingulate cortex (ACC) (Pelgrim, Ramaekers, Wall, Freeman, & Bossong, 2023). This might explain how cannabinoids cause psychotic symptoms by changing connectivity in the SN (Bloomfield et al., 2019).

Twin studies, although limited, demonstrate genetic and environmental influences on cannabis use and its impact on brain development. Studies show that cannabis-related decreases in subcortical volumes reflect common familial influences (Pagliaccio et al., 2015). One study found heritability estimates of 44% and 55% for cannabis initiation and problematic use, respectively (Verweij et al., 2010).

However, no research has yet examined the relationship between cannabis use and PLEs in adolescence or early adulthood, and the genetic and environmental contributions to this relationship. Most prior research focused on chronic cannabis use in Western adult populations, often failing to address brain structural network changes and their association with PLEs in youth. Since multiple circuits are implicated in cannabis-induced brain alterations (McManus, Belnap, Kirsch, Ray, & Grodin, 2025; Menon, 2015), this study looks at the SN, a neural circuit strongly linked to psychosis and cannabis sensitivity. Most of the previous research has focused on chronic or heavy cannabis users; thus, we limited our sample to low-to-moderate, non-daily cannabis users to identify early-stage subclinical effects on brain structure and PLEs. This approach avoids potential confounds related to prolonged exposure and concomitant substance use.

Our primary aim was to look at the relationship among PLEs, recreational cannabis use, and SN parameters during a critical stage of brain development. Based on this, we performed two separate linear mixed models (LMMs) to examine the associations between these variables. Additionally, we explored whether the factors derived from SN parameters mediated the relationship between cannabis use and PLEs. Lastly, we aimed to distinguish the genetic and environmental contributions that are shared among cannabis use, PLEs, and SN parameters by conducting cross-twin within-trait (CTWT) and cross-twin cross-trait (CTCT) correlations, followed by univariate and bivariate twin models.

## Methods

### Participants

Participants were 232 healthy twins (116 pairs), recruited for a broader in-house study on brain development and genetic/environmental risk via posters and social media. Inclusion criteria: (1) age 14–24 and (2) native Turkish speaker. Exclusion criteria: current/past psychiatric/neurological disorders; substance use disorder; progressive visual impairment; and IQ < 70. The study was approved by the Bilkent University Ethics Committee and followed the Helsinki Declaration. Informed consent was obtained from all participants; for minors, parental consent was also secured.

### Measurement

The Community Assessment of Psychic Experiences (CAPE-42) was used to evaluate PLEs in the general population (Stefanis et al., 2002). This 42-item self-report scale measures PLEs across negative, depressive, and positive dimensions (Mark & Toulopoulou, 2015). Participants rated the lifetime frequency of these experiences on a 4-point Likert scale, and a total score was calculated by summing all items. Cannabis use was evaluated with the Cannabis Experience Questionnaire (CEQ), which includes 56 items on subjective effects and usage patterns (Barkus & Lewis, 2018). Individuals with fewer than two lifetime uses were categorized as non-users. All others (i.e. those with ≥ 3 lifetime uses) were considered users. To focus on low-to-moderate frequency recreational use, individuals reporting daily cannabis use were excluded. Although daily users may also use cannabis recreationally, we aimed to examine the effects of occasional use on brain connectivity and PLEs. Daily use can affect brain connectivity in different ways, which might make it harder to see the specific impact of occasional use. Therefore, the one participant who reported daily use was excluded from the final analyses.

To assess current intellectual function, the block design and matrix reasoning subtests of the Wechsler Abbreviated Scale of Intelligence II (WASI-II) were administered using standard procedures (Wechsler, 1999).

### MRI acquisition

Image acquisition was performed with a 3 T Siemens Magnetom Trio scanner using 32-channel radio-frequency coils at the Bilkent University National Magnetic Resonance Research Center (UMRAM), Ankara, Turkey. High-resolution T1-weighted structural images were acquired (TE = 3.02 ms; TR = 2600 ms; TI = 900 ms; flip angle = 8°; slice thickness = 1 mm; field of view (FOV) = 256 mm; matrix = 256 × 256 with 176 slices; and voxel size = 1 × 1 × 1 mm^3^). Diffusion-weighted scans were acquired using an echo-planar imaging (EPI) sequence (TR = 10740 ms; TE = 102 ms; FOV = 206 ms; matrix 256 × 256; slice thickness = 2 mm; and *b* value = 1000 s/mm^2^).

### Diffusion MRI imaging

All preprocessing and matrix generation were conducted using the Connectivity Analysis Toolbox (CATO; de Lange, Helwegen, & van den Heuvel, 2023). T1-weighted MRI preprocessing was completed in Freesurfer (Fischl, 2012), while diffusion-weighted images (DWI) were processed with FSL v6.0 (Jenkinson, Beckmann, Behrens, Woolrich, & Smith, 2012), using Topup and Eddy tools to correct for distortions and motion artifacts (Andersson & Sotiropoulos, 2016). B vectors were updated, and b0 volumes were used to compute DWI reference images. After aligning DWI and T1 images, Freesurfer segmentations were registered. Diffusion tensors were estimated using the RESTORE algorithm (Chang, Jones, & Pierpaoli, 2005) and Levenberg–Marquardt optimization (Press et al., 1992). Average values of voxels along all streamlines connecting pairs of areas defined by the Desikan–Killany Atlas were used to determine fractional anisotropy (FA) and mean diffusivity (MD). FA and MD values show the average microstructural integrity of WM pathways that connect the SN nodes to other SN regions. Thus, these values are an aggregate measure of all streamlines from each SN node, but not individual tracts (e.g. anterior cingulum).

### Anatomical parcellation and network reconstruction

The cortex was parcellated into 114 regions (57 per hemisphere) using the Desikan–Killany Atlas to define network nodes. Whole-brain deterministic tractography was conducted via CATO (de Lange et al., 2023) based on the FACT algorithm, using multiple seeds per WM voxel. Tracking was initiated with a step size of 1 mm and continued until either the FA dropped below 0.2, the turning angle between steps exceeded 45°, or the anatomical stopping criteria were met. To exclude biologically implausible tracts, streamlines shorter than 10 mm or longer than 200 mm were discarded. The resulting streamlines between nodes defined a 114×114 weighted connectivity matrix per participant. Network metrics local efficiency (LE) and clustering coefficient (CC) were computed using the Brain Connectivity Toolbox (Rubinov & Sporns, 2010). These were selected because they reflect how effectively local brain regions communicate (LE) and how strongly they are connected to their immediate neighbors (CC). Given the strong mathematical correlation between CC and LE, we report both but acknowledge their conceptual overlap. Such features may be especially relevant for processing and filtering salient information, which may be altered in cannabis-related psychosis risk. Based on prior studies (McManus et al., 2025; Rössler et al., 2020; Schiwy et al., 2022; Zhao et al., 2022), the anterior insula and caudal ACC were included as key SN nodes. These were not used as tractography seeds but were specifically analyzed for their local connectivity profiles.

### Statistical analyses

All statistical analyses were performed in R (R Core Team, 2021). Initial analyses involved simple linear regressions to investigate unadjusted associations between cannabis use and PLEs. Two separate models for PLE frequency and positive PLEs were defined to explore potential relationships before accounting for covariates and hierarchical data structure.

Missing data (4.9%) across all 24 structural network features (including FA, MD, CC, and LE measures) were imputed using multivariate imputation by chained equations (mice package) (Laird & Ware, 1982). Correlation heatmaps were created to evaluate the relationships among SN variables across different brain regions. These heatmaps demonstrated strong correlations, supporting the need for dimensionality reduction through factor analysis to reduce complexity and enable more parsimonious modeling in subsequent analysis.

Factor analysis was performed to identify the latent constructs reflecting shared variance among SN parameters. The Kaiser–Meyer–Olkin (KMO) measure confirmed the suitability of the dataset for factor analysis (KMO = 0.68). Parallel analysis, a widely used method for determining factor retention (Horn, 1965), indicated the presence of six factors. Although the test for the sufficiency of six factors showed a significant result (χ^2^ = 728.05, df = 319, *p* < 0.001), models with more factors did not provide meaningful improvement. Therefore, the six factors were used as predictors for subsequent analyses. Model performance was assessed using likelihood ratio tests (LRT) and R^2^ metrics. Recent studies have effectively extracted biologically significant components using similar methodologies (Chamberland et al., 2019; Geeraert, Chamberland, Lebel, & Lebel, 2020; Kang, Galdo, & Turner, 2022; Vaher et al., 2022).

These six latent factors were simultaneously entered as fixed effects into an LMM predicting CAPE scores, while adjusting for age, sex, IQ, and cannabis use. A random intercept for family ID was included to account for familial clustering among twin pairs. Of the six latent factors, only Factor 6 was statistically significant (*p* = 0.0339). This factor was mainly influenced by the CC and LE of the left rostral and caudal anterior cingulate cortices, suggesting that Factor 6 represents local connectivity in these regions (Supplementary Table S1). An LRT comparing the full model (with all six factors) to a reduced model (retaining only significant factors) indicated no significant loss of fit (*p* > 0.05), justifying the use of a more interpretable and statistically efficient model in subsequent analyses. Another LRT confirmed that including a random intercept for family ID significantly improved the model fit (χ^2^ (1) = 32.6, *p* < 0.001).

Full model diagnostics, including tests for multicollinearity, interaction effects, assumption checks, and performance metrics, are provided in the Supplementary Materials (Supplementary Figures S2–S3).

### Twin model analyses

Classical twin models use monozygotic (MZ) and dizygotic (DZ) twins to estimate genetic and environmental influences on a single trait or the overlap between traits (Shakoor et al., 2015). While DZ twins share ~50% of additive genetic variance, MZ twins share 100%, along with equal common environmental variance. The CTWT correlations assess the extent to which genetic and environmental factors contribute to the variance in each trait. The univariate twin model further decomposes the variance in a trait into additive genetic (A), common environmental (C), and unique environmental factors (E). A represents the impact of genes that add up to change phenotype and are quantified as narrow-sense heritability (h^2^), representing the proportion of total phenotypic variance attributable to additive genetic effects. C represents nongenetic factors shared by members of the same family and are quantified as c^2^, representing the proportion of variance in a trait due to common environmental influences. E represents environmental components that differentiate members of the same family and are quantified as e^2^, representing the proportion of variance in a trait due to unique environmental influences.

On the other hand, the CTCT reveals whether common genetic and environmental influences contribute to the covariation between two phenotypes (de Vries, van Beijsterveldt, Maes, Colodro-Conde, & Bartels, 2021). Moreover, using bivariate twin models, we can estimate the shared genetic (*r*_g_), common environmental (*r*_c_), and unique environmental (*r*_e_) correlations between two different traits. Furthermore, we can investigate the overall phenotypic correlation between the two traits (*r*_ph_) and decompose this into its additive genetic (*r*_ph-a_), shared environmental (*r*_ph-c_), and unique environmental (*r*_ph-c_) components.

To evaluate the heritability estimates of PLEs, structural connectivity measures, and recreational cannabis use, we first applied CTWT correlations, followed by univariate twin models. Next, we calculated the CTCT correlations, followed by bivariate models to see if PLEs and SN factors shared common genetic and environmental influences with cannabis use.

All twin analyses were conducted using the OpenMx package version 2.20.6 (Boker et al., 2011; Neale et al., 2016) in R version 4.2.2 (R Core Team, 2021). Before the twin analyses, all continuous variables were adjusted for the effects of age, sex, and IQ. All the residuals were standardized, and a saturated model was fitted to the data. For the binary cannabis use variable, age, IQ, and sex were added as covariates to the univariate twin model. To better evaluate the variance estimates of cannabis use, an additional extended model was conducted, including other established environmental risk factors for psychosis, such as childhood trauma, socioeconomic status, and alcohol use as covariates. The *χ* ^2^ goodness-of-fit and Akaike information criterion (Akaike, 1974) were used to evaluate the model fit. The h^2^, c^2^, and e^2^ were calculated along with the likelihood-based 95% confidence intervals.

## Results

### Results of linear regression and linear mixed models

Of 232 individuals, 15 were excluded due to missing data, resulting in a final sample of 217 (59.9% females). Among them, 62 (28.6%) reported recreational cannabis use, while 155 (71.4%) did not. None of the users reported daily consumption; cannabis use was occasional. The average age of onset was 18.26 years (SD = 2.27). Table 1 shows descriptive statistics.

**Table 1.** Demographic characteristics of study participants

	n	%
Count	217	–
Age	19.84 (Mean)	2.43 (S.D)
Sex		
Female	130	59.9
Male	87	40.1
Zygosity		
Monozygotic	85	39.2
Dizygotic	132	60.8
Cannabis experience		
Experienced	62	28.6
Not experienced	155	71.4
The frequency of cannabis use*		
Daily use	0	0
More than once a week	10	4.6
A few times per month	15	6.9
Several times a year	17	7.8
Once or twice	11	5.1
Missing	164	75.6

	Mean	S. D
Psychotic like experiences (CAPE–42)		
Total cape score	72.33	15.99
Positive dimension	29.56	6.06
IQ	102.04	13.56
Age of onset (cannabis)	18.26	2.27

* Cannabis use and frequency were assessed using two separate items from the CEQ: one assessing whether participants had ever used cannabis (yes/no), and another assessing frequency of use. While 62 participants reported having used cannabis at least once, only 53 provided frequency information. The remaining 9 users declined to answer the frequency question, resulting in a higher number of missing responses in the frequency item (n = 164), despite having reported cannabis use experience.

Initial linear regression analyses revealed that cannabis use was significantly associated with higher PLE frequency (β̂ = 5.02, *p* = 0.04), but not with positive PLEs (*p* = 0.05). Age, sex, and IQ were not significant predictors in either model.

To investigate these associations further, LMMs were conducted for both PLE frequency and positive PLEs. In these models, both recreational cannabis use and SN parameters were included as predictors. The SN parameters were modeled as six latent factors obtained from factor analysis, which was used to minimize dimensionality and capture underlying variation across multiple SN regions (Supplementary Table S1). Correlation heatmaps demonstrate the clustering of SN variables across brain regions, which informed the factor extraction (Supplementary Figure S1).

In the model predicting PLE frequency (Model 1), cannabis use (β̂ = 5.39, *p* = 0.029) and SN factor 6 (β̂ = 1.96, *p* = 0.037) emerged as significant predictors. This SN Factor 6 reflects connectivity within the anterior insula and ACC, key SN regions implicated in salience attribution and psychosis risk.

Family-level clustering explained 52% of the variance in PLE frequency (ICC = 0.52), and the inclusion of random intercepts for family ID significantly improved model fit (LRT χ^2^ = 32.62, *df* = 1, *p* < 0.001) (Table 2). For positive PLEs, both cannabis use (β̂ = 2.08, *p* = 0.032) and SN Factor 6 (β̂ = 0.76, *p* = 0.043) were significant predictors. Family-level clustering accounted for 45% of the variance in positive PLEs (ICC = 0.45). The inclusion of a random intercept for family ID significantly improved model fit (LRT χ^2^ = 22.43, *df* = 1, *p* < 0.001) (Table 3).

**Table 2.** Summary of LMM for total CAPE

Predictors	Total CAPE			
	Estimates	Std. Error	CI	p
(Intercept)	59.63	12.77	34.46–84.80	**<0.001**
Sex [male]	−3.49	2.21	−7.84 – 0.86	0.116
Age	−0.06	0.54	−1.12 – 0.99	0.906
IQ	0.14	0.08	−0.02 – 0.30	0.097
Cannabis [1]	5.39	2.46	0.54–10.23	**0.029**
Factor 6	1.96	0.93	0.11–3.80	**0.037**
**Random effects**				
σ^2^	118.62			
τ_00 famid_	128.77			
ICC	0.52			
N _famid_	112			
Observations	217			
Marginal R^2^/conditional R^2^	0.056/0.547			

The bold values indicate statistically significant results at *p* < 0.05.

**Table 3.** Summary of LMM for positive dimension

Predictors	Positive dimension			
	Estimates	Std. Error	CI	p
(Intercept)	29.04	4.87	19.44–38.63	**<0.001**
Sex [male]	−1.04	0.86	−2.74 – 0.67	0.232
Age	0.01	0.20	−0.39 – 0.41	0.974
IQ	0.00	0.03	−0.06 – 0.06	0.947
Cannabis [1]	2.08	0.96	0.18–3.97	**0.032**
Factor 6	0.76	0.37	0.02–1.50	**0.043**
**Random effects**				
σ^2^	19.93			
τ_00 famid_	16.33			
ICC	0.45			
N _famid_	112			
Observations	217			
Marginal R^2^/conditional R^2^	0.038/0.471			

The bold values indicate statistically significant results at *p* < 0.05.

### Mediation analyses

#### Mediation analysis for PLEs frequency

To investigate the mediating role of the SN in the association between cannabis use and PLE frequency, we conducted an exploratory causal mediation analysis using the *mediation* package (Tingley, Yamamoto, Hirose, Keele, & Imai, 2014). Based on prior LMM results, only Factor 6 was included as the mediator due to its significant association with PLEs. Age, sex, and IQ were included as covariates in both the mediator and outcome models to minimize potential confounding.

In the initial mediation model, Factor 6 did not mediate the relationship between cannabis use and PLE frequency (ACME = −0.4054, 95% CI [−1.4606, 0.16], *p* = 0.20). However, the average direct effect (ADE) was significant (ADE = 5.5660, 95% CI [1.0600, 10.34], *p* = 0.04), as was the total effect (total effect = 5.1606, 95% CI [0.4023, 10.00], *p* = 0.04).

A multilevel mediation analysis, accounting for familial clustering, was also conducted using mixed-effects models with cannabis use as a fixed effect and family ID as a random intercept. The outcome model included other covariates as fixed effects. The analysis revealed that ACME was not statistically significant (ACME = −0.470, 95% CI [−1.424, 0.20]), suggesting no evidence of mediation through the SN. However, ADE was significant (ADE = 5.450, 95% CI [1.062, 10.06], *p* = 0.02), indicating a direct relationship between cannabis use and PLE frequency. Similarly, the total effect was significant (total effect = 4.980, 95% CI [0.101, 9.77], *p* = 0.04).

#### Mediation analysis for the positive PLEs

Similarly, we investigated whether SN connectivity mediated the relationship between cannabis use and positive PLEs. The initial mediation analysis using a linear regression model showed no significant mediation through the SN (Factor 6) (ACME = −0.125, 95% CI [−0.400, 0.10], *p* = 0.22). However, cannabis use demonstrated a significant direct effect on positive PLEs (ADE = 2.045, 95% CI [0.641, 3.75], *p* < 0.001), as well as a significant total effect (total effect = 1.919, 95% CI [0.573, 3.56], *p* < 0.001).

These findings were confirmed in the multilevel mediation analysis, which took into consideration family clustering. Cannabis use showed a significant direct effect on positive PLEs (ADE = 5.450, 95% CI [1.062, 10.06], *p* = 0.02) and total effect (Total Effect = 4.980, 95% CI [0.101, 9.77], *p* = 0.04). However, SN Factor 6 did not mediate the effect of cannabis use (ACME = −0.470, 95% CI [−1.424, 0.20], *p* = 0.14).

### Bivariate twin analysis

To assess the underlying etiology of the phenotypic associations identified in the LMMs, we next used classical twin modeling to examine whether recreational cannabis use, PLEs, and SN parameters share genetic or environmental influences. Univariate analysis results (Supplementary Table S2) showed that recreational cannabis use was predominantly affected by environmental factors (h^2^ = 0.00 [−0.61–0.50]; c^2^ = 0.47 [0.04–0.88]; e^2^ = 0.53 [0.37–0.79]). To address the potential role of additional environmental risk factors, we ran an extended univariate model including childhood trauma, alcohol use, and socioeconomic status as covariates. After accounting for covariates, the model indicated unique environmental influences on cannabis use (h^2^ = 0.05 [−1.50–1.35]; c^2^ = 0.44 [−0.67–1.45]; e^2^ = 0.56 [0.17–1.14]). Furthermore, PLE frequency (h^2^ = 0.30 [−0.20–0.81]; c^2^ = 0.29 [−0.17–0.66]; e^2^ = 0.41 [0.27–0.61]) and positive PLEs (h^2^ = 0.45 [−0.16–1.04]; c^2^ = 0.06 [−0.45–0.51]; e^2^ = 0.49 [0.33–0.73]) were influenced by unique environmental factors. Then, we calculated CTCT correlations to investigate the covariance among cannabis use, PLEs, and SN factors derived from the DWI analysis. PLE frequency, positive PLEs, and the SN factors did not show any significant correlations with cannabis use (Table 4). Similarly, no significant phenotypic covariations were found among cannabis use, PLEs, and brain structural connectivity based on the bivariate models (Table 5).

**Table 4.** Cross-twin/sibling cross-trait (CTCT) correlations among cannabis use, CAPE-42 measures, and six factors derived from the salience network

Trait 1	Trait 2	Cross-twin/sibling cross-trait correlations	
		Monozygotic (MZ)	Dizygotic (DZ)
CAPE–42 Total frequency	Cannabis use	0.06 (−0.22/0.34)	**−**0.02 (−0.25/0.22)
CAPE–42 Positive frequency	Cannabis use	0.11 (−0.17–0.38)	−0.15 (−0.37–0.08)
Factor 1	Cannabis use	0.01 (−0.29/0.31)	0.18 (−0.06/0.41)
Factor 2	Cannabis use	−0.12 (−0.41/0.19)	0.07 (−0.18/0.31)
Factor 3	Cannabis use	0.08 (−0.23/0.37)	−0.03 (−0.27/0.21)
Factor 4	Cannabis use	0.24 (−0.07/0.50)	0.02 (−0.23/0.26)
Factor 5	Cannabis use	0.04 (−0.27/0.33)	−0.02 (−0.26/0.22)
Factor 6	Cannabis use	0.02 (−0.29/0.32)	−0.00 (−0.24/0.24)

**Table 5.** Bivariate phenotypic correlations among cannabis use, CAPE-42, and DWI factors obtained from the salience network

Trait 1	Trait 2	*r*_ph-a_	*r*_ph-c_	*r*_ph-e_	*r*_ph_	*r*_g_	*r*_c_	*r*_e_
CAPE–42 total frequency	Cannabis Use	0.17 (−0.19/0.51)	−0.02 (−0.31/0.31)	0.05 (−0.09/0.21)	0.20 (0.00/0.38)	1 (−1/1)	−0.04 (−1/1)	0.13 (−0.28/0.48)
CAPE–42 positive frequency	Cannabis use	0.15 (−0.24/0.45)	−0.07 (−0.30/0.27)	0.11 (−0.04/0.28)	0.20 (0.00/0.37)	1 (−1/1)	−1 (−1/1)	0.31 (−0.11/0.64)
Factor 1	Cannabis use	0 (−0.32/0.31)	0.04 (−0.27/0.31)	−0.19 (−0.40/0.01)	−0.15 (−0.33/0.03)	1 (−1/1)	1 (−1/1)	−0.39 (−0.77/0.01)
Factor 2	Cannabis use	−0.01 (−0.28/0.21)	−0.03 (−0.26/0.19)	−0.15 (−0.37/0.06)	−0.20 (−0.39/0.00)	−1 (−1/1)	−1 (−1/1)	−0.27 (−0.66/0.11)
Factor 3	Cannabis use	0.11 (−0.14/0.35)	0.06 (−0.14/0.29)	−0.03 (−0.21/0.18)	0.13 (−0.04/0.31)	1 (−1/1)	1 (−1/1)	−0.05 (−0.47/0.30)
Factor 4	Cannabis use	0 (−0.23/0.35)	0.14 (−0.05/0.32)	−0.11 (−0.31/0.07)	0.02 (−0.15/0.20)	0.39 (−1/1)	0.60 (−1/1)	−0.21 (−0.52/0.12)
Factor 5	Cannabis use	0 (−0.13/0.13)	−0.05 (−0.19/0.24)	0 (−0.23/0.35)	0.07 (−0.09/0.24)	1 (−1/1)	−1 (−1/1)	0.13 (−0.16/0.42)
Factor 6	Cannabis use	0 (−0.10/0.28)	0.15 (−0.02/0.30)	0.07 (−0.10/0.26)	0.21 (0.00/0.38)	0.21 (−1/1)	1 (−1/1)	0.12 (−0.18/0.42)

*Note:* r_ph-a_, overall additive genetic correlation between two traits; r_ph-c_, overall shared environmental correlation between two traits; _rph-e_, overall unique environmental correlation between two traits; r_c_, common environmental correlations between two traits; r_e_, unique environmental correlations between two traits.

## Discussion

This study examined whether recreational cannabis use during adolescence and young adulthood, a key period for brain development, is linked to PLEs. Additionally, we aimed to disentangle the genetic and environmental influences shared among cannabis use, PLEs, and SN parameters using twin models. While our association analyses examine whether cannabis use is related to PLEs and SN alterations, our twin modeling evaluates whether these associations reflect underlying genetic or shared environmental liability. By combining both approaches within the same sample, we aimed to provide a more comprehensive understanding of the etiology of cannabis-related PLEs during adolescence.

We found a significant association between cannabis use and PLE frequency, suggesting that users report more frequent PLEs than non-users. Although the association with the positive PLEs subscale did not achieve significance in the simple linear regression model (*p* = 0.05), it achieved significance in the LMM after accounting for SN components and familial clustering. Consequently, we approach the result with caution and do not deem it conclusive. Moreover, we found that SN parameters significantly relate to both PLE frequency and positive PLEs, suggesting that SN characteristics may independently contribute to PLE variability. These findings offer preliminary support for the involvement of SN characteristics in PLEs and suggest that cannabis use and SN alterations may represent independent pathways contributing to PLEs. While these findings contribute to current evidence on adolescent cannabis use and susceptibility to psychosis, alternative hypotheses, such as self-medication and pre-existing vulnerabilities, remain plausible.

Another key aim was to test whether SN factors mediate the link between recreational cannabis use and PLEs, given prior evidence of cannabis-related SN disruption, particularly in the ACC and insula. Although some SN factors predicted PLEs, they did not mediate cannabis effects, suggesting the SN may influence PLEs but does not explain the cannabis–PLEs relationship in this sample. To the best of our knowledge, only one other study has investigated whether structural changes in the brain mediate the link between cannabis use and PLEs and found that cannabis use throughout adolescence has no detrimental effect on brain development (DeLisi et al., 2006). Another study found that daily cannabis use was not linked to adverse brain changes, while alcohol use negatively affected brain structure in both adolescents and adults (Weiland et al., 2015). Other studies have also suggested a harmful effect of alcohol, but not cannabis, on the brain (Thayer et al., 2017). Our focus on non-daily users may explain discrepancies with studies including chronic users. Evidence from recent studies suggests that cannabis use does not impair WM integrity (Cousijn, Toenders, van Velzen, & Kaag, 2022; Francis, Camprodon, & Filbey, 2023), a finding supported by a meta-analysis of 830 individuals (Lorenzetti et al., 2023). However, as most studies are observational, the observed link between cannabis use and psychosis risk may reflect familial confounding rather than a direct causal effect (Schaefer et al., 2021), highlighting the importance of twin studies to disentangle genetic and environmental influences.

Building on the findings from the LMMs, we ran twin models to examine whether the observed relationships among cannabis use, PLEs, and SN parameters were driven by shared genetic or environmental influences. Our findings indicate that, during adolescence, recreational cannabis use is predominantly shaped by environmental factors. Moreover, we did not observe significant phenotypic correlations (R_ph_) between PLEs, SN factors, and recreational cannabis use. While LMMs revealed significant associations among cannabis use, PLEs, and SN characteristics, the bivariate models did not reveal shared additive genetic or environmental influences on these traits. These findings indicate that the LMM-based associations may represent environmentally driven, individual-specific mechanisms that are not shared across our phenotypes; however, we cannot rule out methodological issues.

These findings differ from previous studies that have reported moderate-to-high heritability estimates for chronic cannabis use, as well as a phenotypic correlation between chronic cannabis use and PLEs (Karcher et al., 2019; Nesvåg et al., 2016; Verweij et al., 2010). Our study focused on recreational cannabis use in adolescents and young adults rather than chronic cannabis use, which may account for these differences. Given that recreational cannabis use increases during adolescence, a developmental period characterized by heightened environmental sensitivity, it is important to distinguish different types of cannabis consumption. Notably, our findings align with prior research investigating the relationship between adolescent cannabis use and psychoticism. Schaefer et al. (2021), using a longitudinal twin-control design, found no link between adolescent cannabis use and later psychoticism. Our findings from the twin modelling align with this, as the absence of significant phenotypic correlations suggests that cannabis use and PLEs may reflect distinct environmental influences rather than direct phenotypic overlap.

This study contributes novel insights into recreational cannabis use during adolescence and young adulthood by linking it to PLEs and the SN characteristics and emphasizing the role of environmental factors during this key developmental period (Picchioni et al., 2017). Our findings highlight that environmental influences are present in cannabis use among adolescents, an area that has not been extensively explored. We extend previous research by examining covariation among cannabis use, PLEs, and structural brain measures, particularly within a non-Western adolescent and young adult population, a research area and population that is underrepresented in the literature (Batalla et al., 2013).

Several limitations must be considered when evaluating our results. First, although our sample was larger than some previous studies, it was still relatively modest in size. However, the study assessed brain data on young twins, which is a particularly challenging and valuable sample to recruit. Second, cannabis use was assessed via self-report rather than biological verification (e.g. blood or urine tests). Third, cannabis use was treated as a binary variable (users/non-users), without accounting for age of onset, duration, or frequency. Although this approach was consistent with our focus on occasional recreational use, it limits interpretability regarding cumulative exposure. Prior research has shown that WM alterations associated with cannabis use often localize to similar tracts across adolescents and adults (Baker, Yücel, Fornito, Allen, & Lubman, 2013), and exposure duration has been linked to DTI changes (Gruber, Dahlgren, Sagar, Gönenç, & Lukas, 2014; Jacobus & Tapert, 2014; Zalesky et al., 2012). Thus, future studies should use dimensional cannabis measures. However, in the current investigation, these factors were not further investigated due to significant missingness in the relevant questionnaire items, which would have risked statistical validity and decreased model reliability. Furthermore, our sample did not include individuals with daily or heavy cannabis use, nor those with substance use disorders. Rather, it focused on occasional, non-clinical recreational users. This distinction from studies involving chronic or everyday users enabled us to focus on early-stage effects and overcome confounding factors associated with long-term exposure. While this allowed us to investigate early subclinical effects, it limits generalizability to populations with heavier or problematic use, who may exhibit more pronounced alterations in brain structure and psychosis risk (Batalla et al., 2013; Bloomfield et al., 2019). Moreover, we focused on areas relevant to salience processing because earlier work has linked this to cannabis use and psychosis; other brain networks, such as DMN, might have produced different results. Although mediation analyses offered insights into potential pathways linking cannabis use and PLEs, these results should be interpreted cautiously. Causal mediation methods rely on strong assumptions, particularly the assumption of sequential ignorability, which is unlikely to be fully met in observational studies. Thus, results are exploratory and hypothesis generating. Longitudinal studies are needed to more rigorously evaluate these pathways. Although we did not explicitly test the equal environments assumption, we ran an extended model that included key environmental covariates for cannabis use. After adjusting for these variables, the unique environmental influences remained significant, supporting the role of non-shared environmental factors in cannabis use. Nevertheless, other variables, such as cannabis onset age, could still influence variance estimates. Additionally, the variation due to unique environmental influences in twin models also includes measurement errors. Future studies should examine SN functional connectivity to gain more insight into dynamic brain processes potentially affected by cannabis usage.

In conclusion, our findings indicate that adolescent and early adulthood recreational cannabis use is associated with an increased risk of PLEs, which are primarily influenced by environmental factors rather than genetic predisposition. These symptoms may be transient and have no clear evidence of lasting changes in brain networks like the SN.

## Supporting information

Atmaca-Turan et al. supplementary material Atmaca-Turan et al. supplementary material

## Data Availability

All statistical analyses were performed in R. While the full analysis code is not publicly hosted at this stage, it is available from the corresponding author upon request for verification or reproduction of results.

## Supplementary material

The supplementary material for this article can be found at http://doi.org/10.1017/S0033291725101773.
