# Supplementary material for: The relationship between recreational cannabis use, psychotic-like experiences, and the salience network in adolescent and young adult twins
Source: Psychol Med. 2025 Oct 7;55:e300. doi: 10.1017/S0033291725101773 (PMC12527529; doi:10.1017/S0033291725101773)
Supplement: Atmaca-Turan et al. supplementary material [file S0033291725101773sup001.docx]

**SUPPLEMENTARY MATERIAL**

**Linear Regression and Linear Mixed Models**


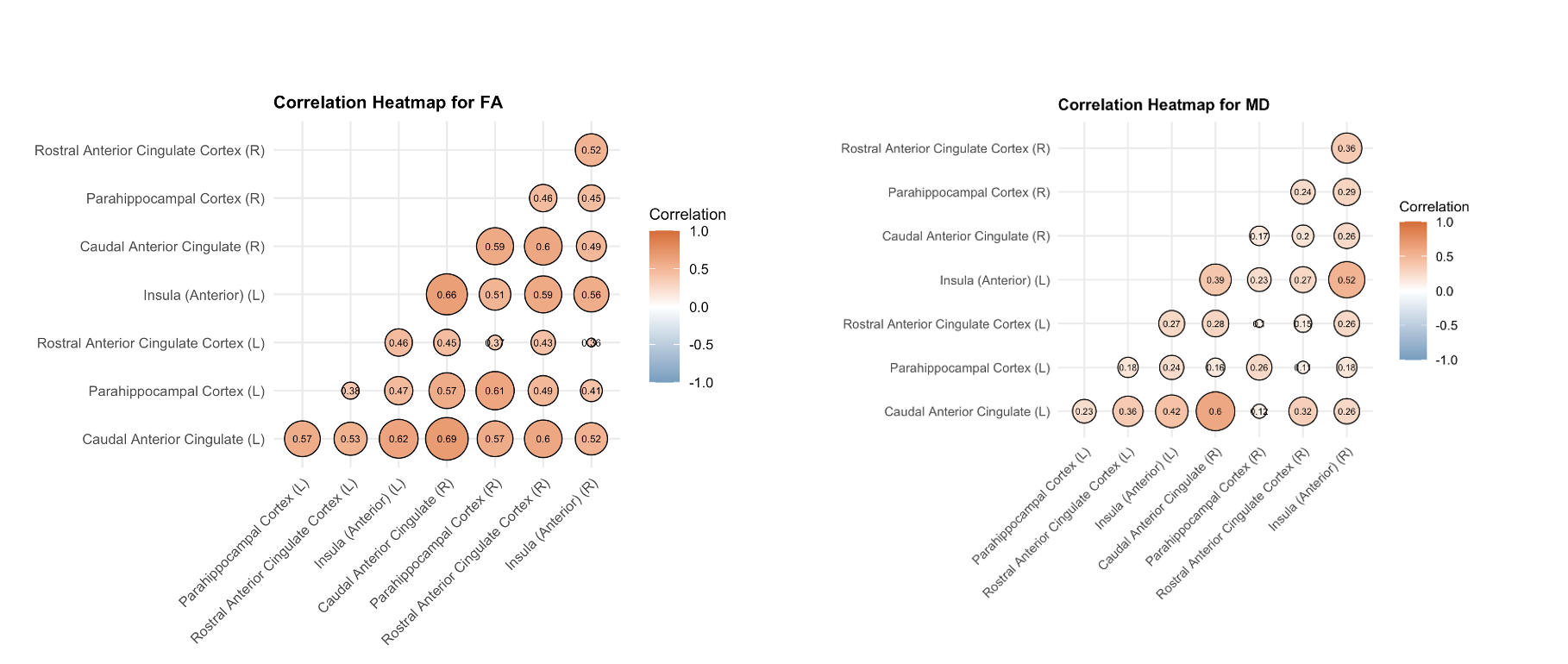

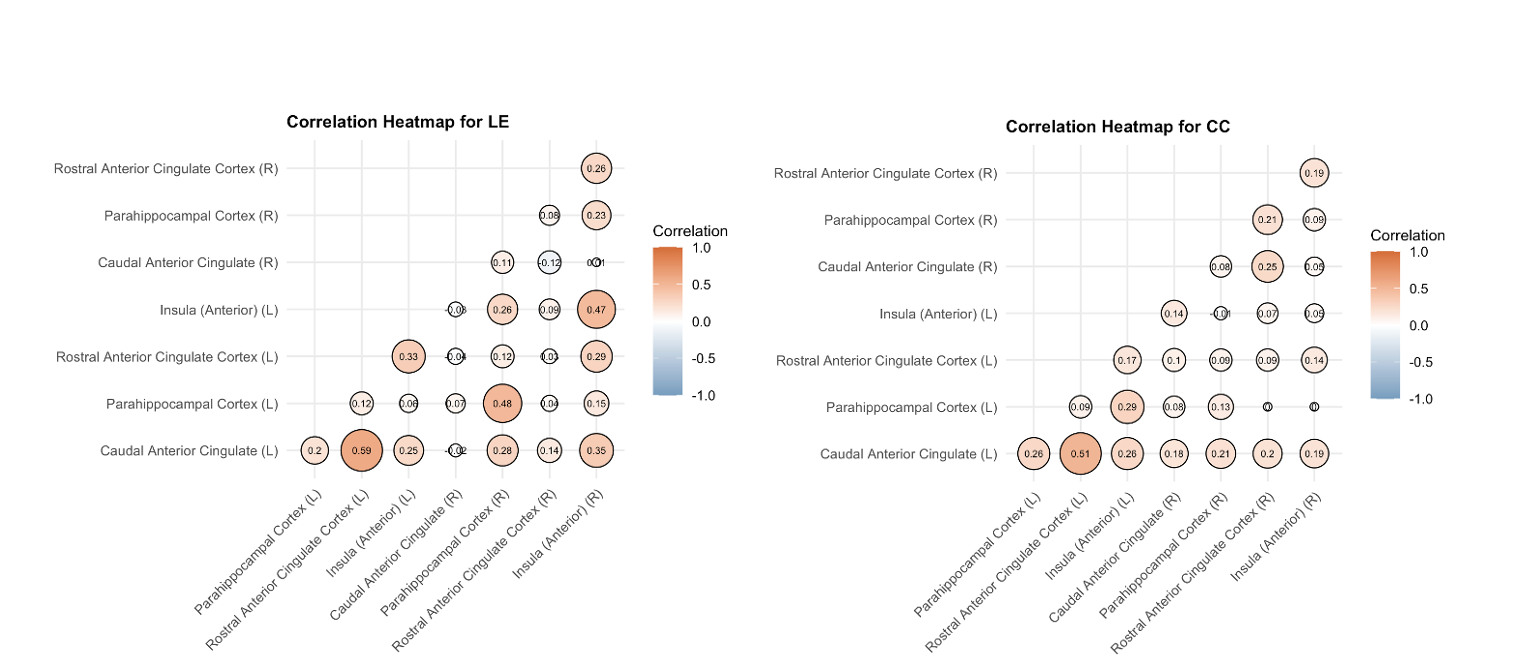
**Figure S1** Correlation heatmaps presented illustrate the clustering of salience network variables across brain regions, which informed the construction of these factors.

***Abbreviations:*** FA: fractional anisotropy; MD: mean diffusivity; CC: clustering coefficient; LE: local efficiency; R: right; L: left

**Model Diagnostics**

All models were assessed for multicollinearity using variance inflation factors (VIFs), with all values below the conservative threshold of 5. We also tested for potential interactions between cannabis use and the significant latent factor by adding interaction terms individually, but none reached statistical significance. The same modeling framework was applied to examine associations with the **CAPE Positive Dimension,** following identical steps for model specification, selection, and diagnostics.

Model diagnostic assessments confirmed adequate fit for our analyses. Diagnostic plots for the total CAPE scores model (Figure S2) revealed no violations of key statistical assumptions. For the CAPE Positive Dimension model, one observation displayed a standardized residual below -3, indicating a potential outlier. However, given that this observation represents a legitimate data point and its removal did not substantially alter the conclusions in sensitivity testing, we retained all observations in our final analyses. Diagnostic plots (Figure S3) demonstrated that model assumptions were also satisfied for the CAPE Positive Dimension model, supporting the robustness of our findings.

**Figure S2** Model diagnostic plots for the regression model predicting total CAPE scores. The plots confirm that model assumptions (linearity, homoscedasticity, normality of residuals, and absence of influential outliers) were adequately met.

**
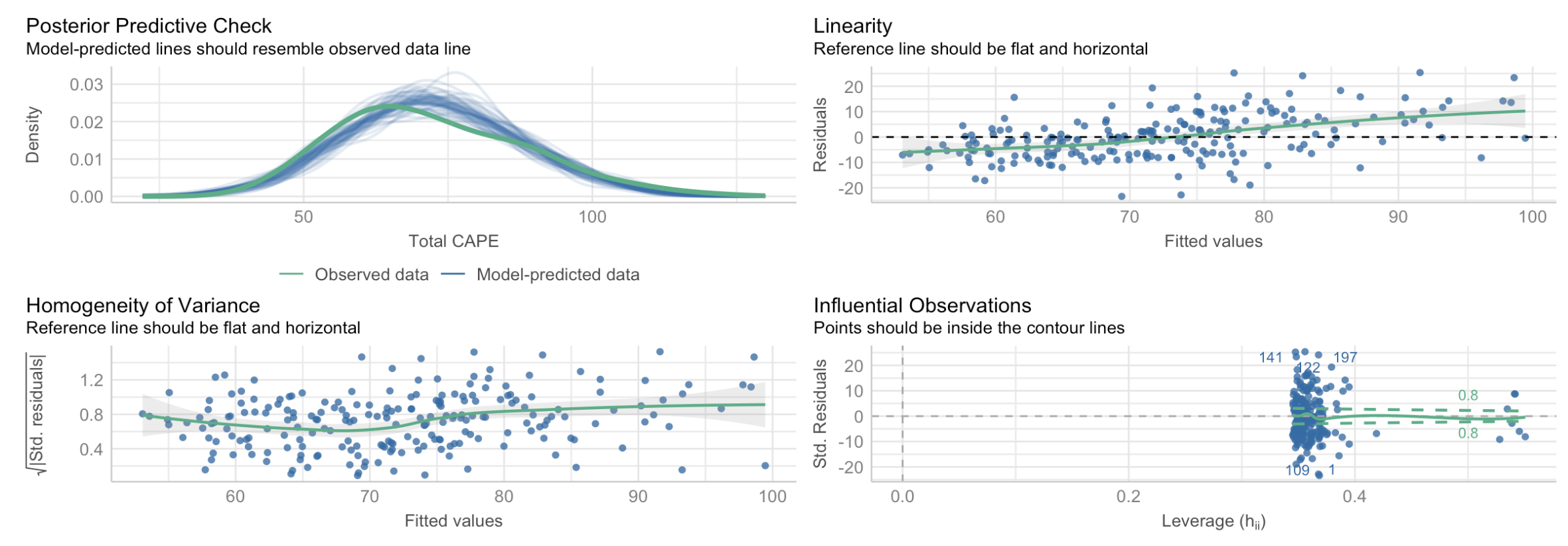
**

**Figure S3** Model diagnostic plots for the regression model predicting CAPE positive dimension scores. One observation (row 61) had a standardized residual less than -3. The model was refitted after excluding this observation, and the results remained consistent. The diagnostic plots indicate that model assumptions were generally met.

**
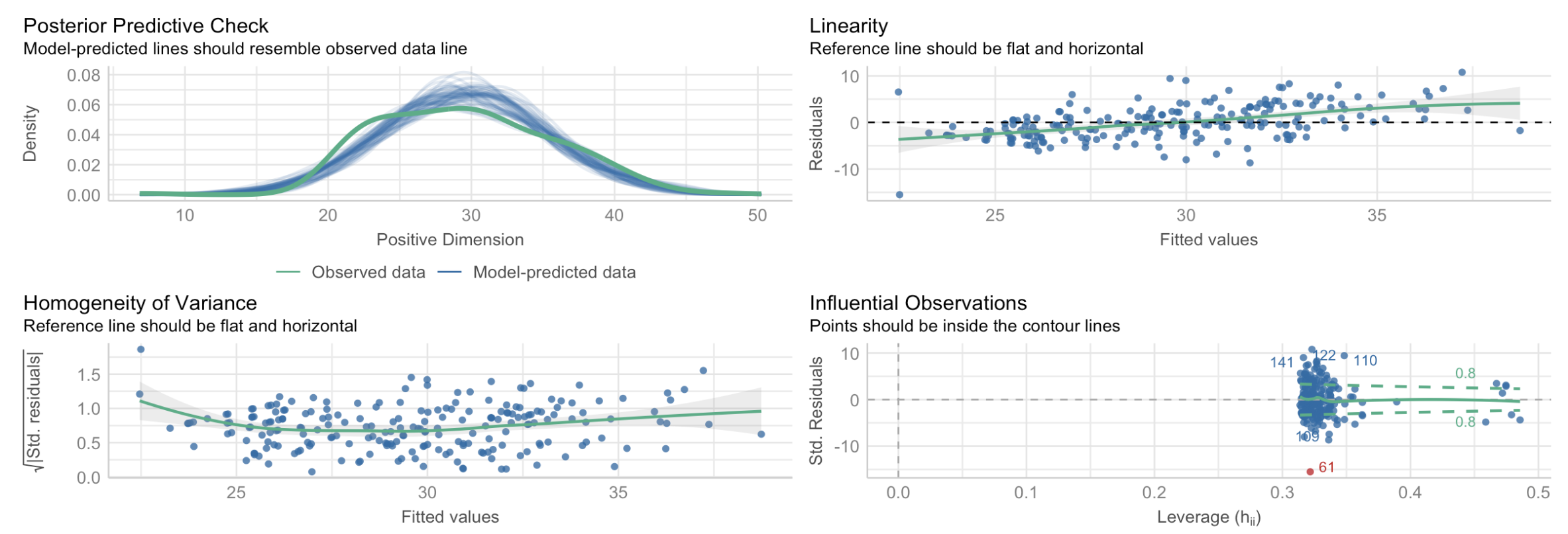
**

**Factor Loadings**

**Supplementary Table 1 (S1)**

| Regions | Factor1 | Factor2 | Factor3 | Factor4 | Factor5 | Factor6 |
| --- | --- | --- | --- | --- | --- | --- |
| cACC_FA (L) | **0.81640443** | -0.0533637 | -0.046872 | -0.0099444 | **0.127966** | 0.0361667 |
| PHC_FA (L) | **0.68993005** | -0.0096155 | 0.01805044 | 0.05151296 | -0.0675746 | 0.06656389 |
| rACC_FA (L) | **0.56106421** | -0.0157769 | **-0.1420489** | **-0.1035829** | **0.10926432** | -0.0556916 |
| aINS_FA (L) | **0.77198864** | 0.07081085 | -0.0388865 | -0.048031 | 0.01274012 | 0.04314615 |
| cACC_FA (R) | **0.81588617** | 0.0538668 | -0.0684015 | 0.01847061 | 0.02400393 | 0.04654855 |
| PHC_FA (R) | **0.70698642** | -0.0125834 | 0.03272988 | 0.04665595 | -0.0755509 | 0.01919457 |
| rACC_FA (R) | **0.74455031** | -0.0192881 | **-0.1364762** | -0.0488617 | 0.04736392 | -0.0173 |
| aINS_FA (R) | **0.65015707** | **-0.157779** | 0.02000474 | -0.067953 | 0.06351106 | -0.0173279 |
| cACC_MD (L) | 0.07469491 | **0.63904325** | -0.0002655 | 0.08945247 | 0.09669123 | **0.12585249** |
| PHC_MD (L) | 0.05023264 | **0.34803449** | **-0.114629** | -0.0188525 | 0.00165906 | -0.008115 |
| rACC_MD (L) | 0.02929773 | **0.42891517** | 0.00360298 | 0.06619376 | 0.02682888 | 0.06664189 |
| aINS_MD (L) | 0.0486058 | **0.68720157** | 0.06966876 | 0.0416517 | **0.15973847** | 0.03753735 |
| cACC_MD (R) | 0.08545235 | **0.58164757** | **0.11821801** | 0.09532336 | -0.0019619 | 0.06016499 |
| PHC_MD (R) | -0.0969384 | **0.37148198** | 0.01335792 | 0.01718504 | -0.0538121 | -0.048139 |
| rACC_MD (R) | **-0.2783341** | **0.45823049** | 0.09024932 | -0.0462484 | -0.0061422 | 0.04553247 |
| aINS_MD (R) | **-0.1639701** | **0.61233213** | 0.02689703 | **-0.1311976** | 0.09456334 | -0.0870435 |
| cACC_CC (L) | -0.0005615 | **0.12995395** | **0.20940492** | 0.06010021 | **0.81025956** | **0.28896546** |
| PHC_CC (L) | -0.0396363 | 0.04141056 | 0.07673903 | **0.82870653** | **0.14995243** | 0.03367283 |
| rACC_CC (L) | 0.0671792 | **0.13009491** | **0.19047697** | 0.00426693 | **0.22718303** | **0.94109569** |
| rACC_CC (L) | **-0.126551** | **0.11712829** | **0.51755041** | -0.0060414 | 0.03108548 | 0.05833867 |
| cACC_CC (R) | 5.4044E-05 | 0.00159821 | **0.17018624** | **0.13650184** | 0.09467263 | 0.05065039 |
| PHC_CC (R) | 0.05074359 | 0.02725543 | 0.04785423 | **0.10338128** | **0.202109** | 0.0247318 |
| rACC_CC (R) | 0.07259178 | 0.05713867 | **0.27358809** | -0.009873 | **0.16471012** | -0.0103526 |
| aINS_CC (R) | **-0.1344839** | -0.099727 | **0.28509352** | 0.0278682 | **0.11579451** | 0.08302441 |
| cACC_LE (L) | 0.04348995 | 0.06479841 | **0.27644396** | 0.08813621 | **0.86826234** | **0.24472328** |
| PHC_LE (L) | -0.0105628 | 0.00032356 | 0.05379033 | **0.98941404** | 0.09043321 | 0.06984453 |
| rACC_LE (L) | 0.04520087 | 0.09980474 | **0.24078614** | 0.01843531 | **0.35304647** | **0.79582294** |
| rACC_LE (L) | -0.065235 | 0.01130559 | **0.98139868** | -0.0021262 | -0.0635043 | **0.15308938** |
| cACC_LE (R) | -0.0335615 | 0.02336508 | -0.0355103 | 0.07901374 | -0.0144288 | -0.0132969 |
| PHC_LE (R) | 0.06409435 | -0.0002265 | **0.28991682** | **0.45675846** | **0.17188392** | -0.0513809 |
| rACC_LE (R) | **0.13837355** | 0.06889888 | **0.11872376** | 0.02629114 | **0.11794415** | -0.0853865 |
| aINS_LE (R) | **-0.1012453** | -0.0218753 | **0.4807517** | **0.10088689** | **0.24746672** | 0.0674064 |

***Note.*** L = left; R = right; FA = fractional anisotropy; MD = mean diffusivity; CC = clustering coefficient; LE = local efficiency; rACC = rostral anterior cingulate cortex; cACC = caudal anterior cingulate cortex; PHC = parahippocampal cortex; aINS = anterior insula.

**Univariate Twin Analysis**

The cross-twin/sibling within-trait correlations and the univariate twin models were calculated for cannabis use, CAPE-42 total frequency, its positive subscale, and 6 factors derived from the factor analysis. The results are presented in Supplementary Table 1 (Table S1).

The results of the CTWT correlations showed higher MZ correlations in cannabis use and the positive frequency scale of CAPE-42, suggesting genetic influences on these traits. Additionally, the correlation coefficients were significantly larger in MZ twins compared to DZ twins for the first factor.

Univariate twin models were conducted to estimate the relative contributions of genetic, common, and unique environmental influences to the total variance of a trait. For cannabis use, the ACE model revealed that 47% of the variance was attributed to common environmental influences and 53% to unique environmental influences (h^2^= 0 [-0.61-0.50]; c^2^= 0.47 [0.04-0.88]; e^2^= 0.53 [0.37-0.79]). Positive frequency scale and CAPE-42 total frequency scores were predominantly influenced by unique environmental factors. For the factor analysis, all the factors were predominantly influenced by unique environmental factors.

**Supplementary Table 2 (S2)** Cross-twin/sibling within-trait correlations, and the estimates of h², c², and e²

| **Variable** | **Cross-twin/sibling Within-Trait Correlations** | | **h^2^** | **c^2^** | **e^2^** |
| --- | --- | --- | --- | --- | --- |
|  | **MZ** | **DZ** |  |  |  |
| Cannabis use | **0.44**  (0.18/0.65) | **0.48**  (0.28/0.64) | 0  (-0.61/0.50) | **0.47**  (0.04/0.88) | **0.53**  (0.37/0.79) |
| CAPE-42 | **0.62**  (0.41/0.77) | **0.43**  (0.21/0.60) | 0.30  (-0.20/0.81) | 0.29  (-0.17/0.66) | **0.41**  (0.27/0.61) |
| Positive CAPE | **0.63**  (0.41/0.77) | **0.26**  (0.03/0.47) | 0.45  (-0.16/1.04) | 0.06  (-0.45/0.51) | **0.49**  (0.33/0.73) |
| FAC1 | **0.39**  (0.1/0.63) | 0  (-0.25/0.24) | 0.62  (-0.33/1.55) | -0.33  (-1.17/0.53) | **0.71**  (0.53/0.92) |
| FAC2 | 0.14  (-0.18/0.43) | -0.08  (-0.33/0.17) | 0.46  (-0.33/1.21) | -0.36  (-0.97/0.31) | **0.89**  (0.66/1.14) |
| FAC3 | 0.11  (-0.2/0.4) | -0.08  (-0.32/0.17) | 0.38  (-0.49/1.20) | -0.29  (-0.99/0.48) | **0.91**  (0.69/1.13) |
| FAC4 | -0.07  (-0.37/0.24) | 0.14  (-0.12/0.37) | 0.00  (1.67/0.76) | 0.05  (-0.371/1.08) | **0.95**  (0.55/1.64) |
| FAC5 | -0.17  (-0.45/0.15) | **-0.26**  (-0.48/-0.02) | 0.23  (-0.49/0.96) | -0.30  (-0.91/0.19) | **1.21**  (0.87/1.41) |
| FAC6 | -0.12  (-0.42/0.19) | 0.04  (-0.21/0.28) | 0.00  (-1.13/0.41) | 0.21  (-0.35/0.80) | **0.79**  (0.85/1.41) |

***Note:*** Values in bold denote the significance based on 95% confidence intervals

***Abbreviations*:** MZ = Monozygotic twins; DZ = Dizygotic twins; h² = Heritability estimate; c² = Shared environment estimate; e² = Unique environment estimate; FA: Fractional anisotropy; MD: Mean diffusivity; CC: Clustering coefficient; LE: Local efficiency; L: left; R: right; cACC: Caudal anterior cingulate cortex; mOFC: Medial orbitofrontal cortex; aINS: Anterior insula; PCC: Posterior cingulate cortex.

**Extended Cannabis Use Model with Environmental Covariates**

To adjust for the effects of environmental variables, an extended model was run for cannabis use, which included childhood trauma as measured by the Childhood Trauma Questionnaire (Bernstein et al., 2011; Şar et al., 2021), alcohol use (Robins, 1988), and socioeconomic status as covariates. After accounting for the environmental risk factors, the full ACE model showed significant unique environmental influences on cannabis use (h2= 0.05 [-1.50-1.35]; c2= 0.44 [-0.67-1.45]; e2= 0.56 [0.17-1.14]).

**References**

Bernstein, D. P., Fink, L., Handelsman, L., & Foote, J. (2011). Childhood Trauma Questionnaire. In *PsycTESTS Dataset*. https://doi.org/10.1037/t02080-000

Robins, L. N. (1988). The Composite International Diagnostic Interview. *Archives of General Psychiatry*, *45*(12), 1069. https://doi.org/10.1001/archpsyc.1988.01800360017003

Şar, V., Necef, I., Mutluer, T., Fatih, P., & Türk-Kurtça, T. (2021). A Revised and Expanded Version of The Turkish Childhood Trauma Questionnaire (CTQ-33): Overprotection-Overcontrol As an Additional Factor. *Journal of Trauma & Dissociation*, *22*(1), 35–51. https://doi.org/10.1080/15299732.2020.1760171
